# Selected, Deselected, and Reselected: A Case Study Analysis of Attributes Associated With Player Reselection Following Closure of a Youth Soccer Academy

**DOI:** 10.3389/fspor.2021.633124

**Published:** 2021-03-23

**Authors:** James H. Dugdale, Allistair P. McRobert, Viswanath B. Unnithan

**Affiliations:** ^1^Physiology Exercise and Nutrition Research Group, Faculty of Health Sciences and Sport, University of Stirling, Stirling, United Kingdom; ^2^The Football Exchange, Liverpool John Moores University, Liverpool, United Kingdom; ^3^Research Institute for Sport & Exercise Sciences, Liverpool John Moores University, Liverpool, United Kingdom; ^4^Division of Sport and Exercise, School of Health and Life Sciences, University of the West of Scotland, Hamilton, United Kingdom

**Keywords:** talent identification, talent development, selection, recruitment, football, decision making

## Abstract

Considering the perceived benefit of early recruitment and the time and resources spent developing youth players, individuals released from talent development programmes are often re-recruited by rival academies. However, due to the contractual nature of many talent development programmes, limited empirical data exists on players deselected from (or reselected to) youth soccer academies. Adopting a novel case study approach, differences in skill, psychological, and physical attributes associated with reselection following closure of a junior-elite soccer academy were explored. Overall subjective coach ratings for skill, psychological, and physical abilities; subjective coach ratings for skill and psychological attributes; and physical fitness test performance of 79 junior-elite soccer players (U11–U17) were assessed as part of regular scheduled testing and monitoring practices prior to the academy closure. Reselection status was monitored and recorded for all players in the 6 months following the academy closure and was classified as a persistence/progression (“Reselected”) or attrition (“Deselected”) in playing level. Of the 79 released players, a total of 60 players (76%) were re-signed to a junior-elite academy within 6 months. Differences were observed for overall ratings of skill, psychological, and physical abilities in favor of the “Reselected” player group. “Reselected” players were also rated higher by coaches for all attributes categorized as skill and psychological, as well as performing better at all physical fitness tests. However, “Reselected” players were lesser in stature and body mass and less mature than “Deselected” players. Our findings suggest that reselection is not a product of anthropometric criteria and, therefore, a pathway for selection remains open for later maturing players. We also inform upon desirable qualities associated with player reselection and provide a case study approach of a unique, yet highly relevant, scenario for talent identification and development in youth soccer.

## Introduction

Talent identification (TI) and talent development (TD) of youth players are important factors when considering future financial and competitive benefits for soccer clubs (Unnithan et al., 2012). In soccer, professional clubs operate academy systems providing systematic training programmes for youth players starting as young as age four, and progressing toward professional transition at ~18 years of age. Early recruitment and prolonged exposure to TD programmes is highly desirable for coaches and recruiters, as it provides a greater timeframe to develop skills and expertise necessary to succeed at the professional level (Vaeyens et al., 2008; Burgess and Naughton, 2010; Williams et al., 2020). However, high turnover of youth players is reported within professional soccer academies, with only ~10% of players successful in obtaining professional contracts (Grossmann and Lames, 2015).

It is well-established that potential predictors of talent are multidimensional in nature (Reilly et al., 2000; Unnithan et al., 2012; Sieghartsleitner et al., 2019). Therefore, consideration is given to physical, sociological, and psychological attributes along with technical skill abilities when making decisions around TI and (de)selection in soccer (Williams et al., 2020). Evidence from the extant literature suggest that differences in multidimensional characteristics are evident between distinct playing standards of youth soccer players (Waldron and Worsfold, 2010; Huijgen et al., 2015; Dugdale et al., 2019), and they develop resultant of exposure to TD programmes (Burgess and Naughton, 2010; Williams et al., 2020). However, due to the contracted nature of many TD programmes, limited empirical data exist on players deselected from (or reselected to) youth soccer academies.

In their study of elite Dutch soccer players, Huijgen et al. (2014) suggested that differences in technical, tactical, and physiological characteristics may exist between players selected vs. deselected from TD programmes, supporting the value of multidimensional performance assessments to inform selection decisions in more homogenous groups. Similarly, Figueiredo et al. (2009) found that Portuguese youth soccer players selected to a higher competitive playing level performed better in functional capacities and skills tests comparative to players who persisted or regressed in playing level. Finally, when considering the impact of deselection and reselection on long-term TD, Güllich (2014) found that players who were successful at the professional level experienced repeated selection and deselection through youth, as opposed to early selection and continuous, long-term nurture within German TD programmes, advocating the value of reselecting previously deselected players within TI processes.

Biological and anthropometric factors may also influence TI and (de)selection processes within academy soccer. A plethora of evidence suggests that soccer players selected to academy programmes are larger in body size and biologically more mature compared to players of a lesser playing level (see Malina et al., 2017 for a review). Furthermore, an asymmetry in birthdate distribution favoring those born earlier in the selection year, commonly referred to as the relative age effect (RAE), is widely reported within academy soccer (Helsen et al., 2005; Carling et al., 2009; Lovell et al., 2015). Scientists suggest that these factors (un)consciously affect decision making during TI and TD processes due to acute performance benefits, despite concerns around the value of these attributes to youth-professional transition (Meylan et al., 2010; Kelly and Williams, 2020). However, although the extant literature on deselected and reselected players is scare, evidence suggests that biological, anthropometric attributes or birthdate distributions do not differentiate between these player groups, likely due to the physical and biological homogeneity of academy soccer players (Huijgen et al., 2014; Platvoet et al., 2020).

Considering the influence that financial decisions play on soccer academy operations (Reeves et al., 2018b), several clubs have recently made the decision to close their academies, prioritizing investment in their first team and sourcing players externally as opposed to investing in home-grown talent. In contrast, there are many clubs who greatly value their academies, placing TI and TD at the forefront of their philosophy and operations (Cushion et al., 2012; Larkin and Reeves, 2018; Reeves et al., 2018b). This may result in a core of players who possess the same ethos and an affinity for the club, potentially leading to talented academy graduates or income generation through player sales and transfers (Grossmann and Lames, 2015). Considering this disparity in philosophy and the perceived benefit of systematic TD, clubs invested in youth academy infrastructures may view deselected players favorably. As a result, individuals released from academy programmes are often recruited by rival clubs (Vaeyens et al., 2008; Unnithan et al., 2012). Providing information regarding multidimensional attributes associated with reselection of players deselected from TD systems would, therefore, be valuable for coaches and practitioners working within TI and TD.

Adopting a novel case study design, we explored differences in multidimensional attributes of players related to persistence/progression (“Reselected”) or attrition (“Deselected”) in playing level following closure of a youth soccer academy. Considering the rarity of this scenario, our findings may inform upon desirable qualities associated with player reselection and provide a case study approach of a unique yet highly relevant scenario for talent identification and development in youth soccer.

## Methods

### Participants

A total of 79 male youth soccer players aged 10.2 to 16.7 years (M: 13.2 ± 1.9) were recruited. At the time of data collection, players were affiliated to a “Progressive” junior-elite soccer academy as classified by the “Project Brave” initiative of the Scottish Football Association (SFA) (SFA, 2017). Participants were categorized into age groups as specified by the SFA: U11 (*n* = 16); U12 (*n* = 14); U13 (*n* = 10); U14 (*n* = 12); U15 (*n* = 12); and U17 (*n* = 15). Informed participant assent, parental/guardian consent, and Academy Director gatekeeper consent was gained. The study received institutional ethical approval from the local university ethics board (GUEP 533R).

### Procedures

We used an exploratory case study design (Yin, 2009; Reeves et al., 2019) using players affiliated to a junior-elite soccer academy in Scotland. In December 2017, the club made the decision to close the academy, releasing players from their contracts. Subsequently, between December 2017 and June 2018 players either (a) re-signed with a SFA “Elite” or “Progressive” junior-elite academy (considered as persistence or progression in playing level—“Reselected”) or (b) signed with a SFA “Performance” junior-elite soccer academy, signed with an amateur club, or took a break from playing altogether (considered an attrition in playing level—“Deselected”). We collected physical fitness test, anthropometrics and maturity offset, and subjective coach rating data as part of routine Academy operations in December 2017, prior to the winter break of play. Academy staff monitored players' status and club affiliations for the subsequent 6 months following the academy closure, and the Academy Director provided the authors with these details for all players in June 2018.

#### Fitness Tests

We collected data on five measures of physical fitness using established methods: Yo-Yo Intermittent Recovery Test Level 1 (YYIRT L1; Krustrup et al., 2003); countermovement vertical jump (CMJ; Murtagh et al., 2018); Functional Movement Screen™ (FMS; Cook et al., 2006); and 5 m/20 m linear sprint tests (Enright et al., 2018). Such measures have been applied to samples of youth athletes and are valid and reliable tests (Krustrup et al., 2003; Lloyd et al., 2015; Enright et al., 2018; Dugdale et al., 2019). Moreover, we recorded body mass, standing stature, and seated height. A regression equation was used to provide somatic maturity estimates, presented as maturity offset (years from age at peak height velocity; Mirwald et al., 2002).

Fitness testing for all participants was completed as part of routine testing and monitoring practices by the Academy in December 2017, prior to players being released from their contracts. The fitness testing session was completed a minimum of 48 h following a competitive game and in the absence of strenuous exercise within 24 h prior. Fitness testing was conducted indoors on a non-slip surface with an ambient temperature of ~18°C. All players received the same standardized warm-up consisting of light aerobic activity, dynamic stretching, progressive sprinting, and sub-maximal jump variations. Tests were completed in a standardized order and arranged from least-to-most physically demanding by the research team to manage fatigue (anthropometrics > FMS > CMJ > linear sprint > YIIRT L1). For the linear sprint and CMJ tests, participants completed three attempts with the best attempt for each test being selected for analysis.

#### Coach Ratings

Coaches rated players on 29 multidimensional attributes identified as important to the recruitment process of youth soccer players by Larkin and O'Connor (2017). Coaches used a 5-point Likert scale to rate the attributes of each player relative to their age and stage of development: 1 – *poor*; 2 – *below average*; 3 – *average*; 4 – *very good*; 5 – *excellent*. Such coach-based rating methods have previously been adopted by researchers and they demonstrate good reliability and validity (Unnithan et al., 2012; Fenner et al., 2016; Hendry et al., 2018; Dugdale et al., 2020). Coaches were provided with definitions of the attributes established by Larkin and O'Connor (2017) and allowed to ask questions to the research team prior to assigning their ratings. The research team also provided hypothetical examples to the coaches of what would be considered “poor” or “excellent” for each attribute to ensure clarity. For example, “excellent” “1 v 1” ability was described to coaches as “a player who regularly beats the opposition during an individual match-up leading to progression of position or opportunity for their team, particularly during pressurized situations.” Further, “poor” “concentration” was described as “a player who makes regular errors due to inconsistent mental effort applied during training and competition, often leading to the loss of possession or opponent goal scoring opportunities.”

Coaches also provided an overall category rating for players' “skill,” “psychological,” and “physical” abilities on an identical 5-point Likert scale. The coaches completed their subjective ratings independently without confirmation with the research team, other coaches, or support staff.

#### Attribute Categorization

Models of TI in soccer were identified and reviewed by the research team (JD, AMcR, VU). The Williams et al. (2020) model was selected for implementation in this study due to being the most recently published model. The category of “sociological” identified within the Williams et al. (2020) model was removed resultant of the inability of coaches to appropriately rate sociological attributes. Attributes were then categorized by the research team as either “physical,” “skill,” or “psychological” according to the model of potential predictors of talent in soccer by Williams et al. (2020). Attributes were categorized using a three-stage approach: (1) the research team individually categorized attributes, resulting in unanimous agreement for 24/29 attributes; (2) the research team discussed the remaining attributes electronically (*n* = 5) with reference to the attribute definitions provided by Larkin and O'Connor (2017) and with reference to the Williams et al. (2020) model, resulting in agreement for a further 4 attributes; and (3) the research team met face-to-face to discuss the remaining attribute, resulting in agreement. Following this three-stage process, the research team reached agreement for all 29 attributes. As a consequence of the separately collected, objective fitness test data utilized in this study, attributes categorized into the “physical” category (*n* = 4) were removed prior to analysis. A total of 25 subjectively rated attributes (skill: *n* = 14; psychological: *n* = 11) were included for analysis.

### Statistical Analysis

Considering the exploratory nature of this study, we provide descriptive statistics for “Reselected” and “Deselected” players. “Reselected” and “Deselected” players are reported as frequencies and percentages (%). Subjective ratings are reported as means and standard deviations (SD) for both “Reselected” and “Deselected” player groups. For objective physical measures, test scores were standardized using z-scores ± SD. This involved allocating within-age group standardized scores and collapsing across levels prior to analysis. This allowed for comparisons between “Reselected” and “Deselected” players by removing potential age effects associated with performance due to the disparity of comparison groups. Standardized effect size, reported as Cohen's *d* using the pooled SD as the denominator, was calculated to evaluate the magnitude of difference between the two groups. Qualitative interpretation of *d* was based on the guidelines provided by Hopkins et al. (2009): 0–0.19 trivial; 0.20–0.59 small; 0.60–1.19 moderate; 1.20–1.99 large; ≥2.00 very large.

Birthdates for all players were categorized into the following relative age quartiles from the start of the selection year specified by the SFA (Dugdale et al., 2021): Q1 = Jan–Mar; Q2 = Apr–Jun; Q3 = Jul–Sep; Q4 = Oct–Dec, and reported as frequencies and percentages (%). The Chi squared (χ^2^) test was used to assess differences between observed and expected birthdate distributions across quartiles for both “Reselected” and “Deselected” players. Odds ratios (OR) and 95% confidence intervals (95%CI) were calculated to compare the birthdate distribution of a quartile (Q1, Q2, or Q3) with the reference group, consisting of the relatively youngest players (Q4). Data were analyzed via SPSS Statistics Version 25.0 for Windows (IBM, Chicago, Illinois, USA).

## Results

**Figure 1:**
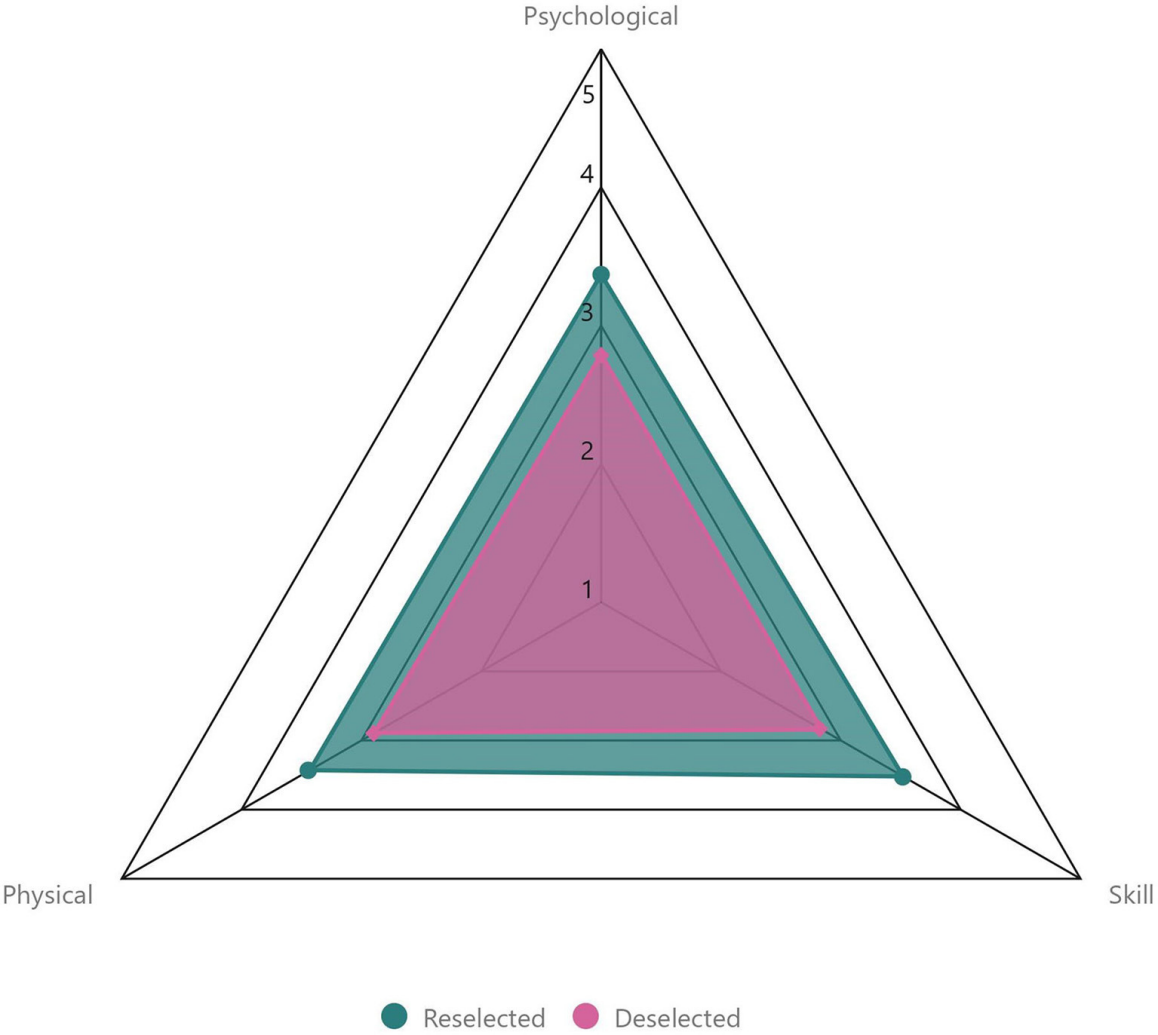
Comparison of overall coach category ratings between “Reselected” and “Deselected” players.

Of the 79 players within our study, a total of 60 players (76%) were “Reselected” in the 6 months following the academy closure. “Reselected” players represented: U11 – *n* = 11/16; U12 – *n* = 11/14; U13 – *n* = 9/10; U14 – *n* = 7/12; U15 – *n* = 9/12; and U17 – *n* = 13/15 of each age group within our sample. Overall subjective coach ratings for the three multidimensional categories were: 3.5 ± 0.8 vs. 2.8 ± 0.8 (Skill); 3.4 ± 0.8 vs. 2.8 ± 1.0 (Psychological); and 3.4 ± 0.9 vs. 2.9 ± 0.9 (Physical) for “Reselected” vs. “Deselected” players, respectively (Figure 1).

**Figure 2:**
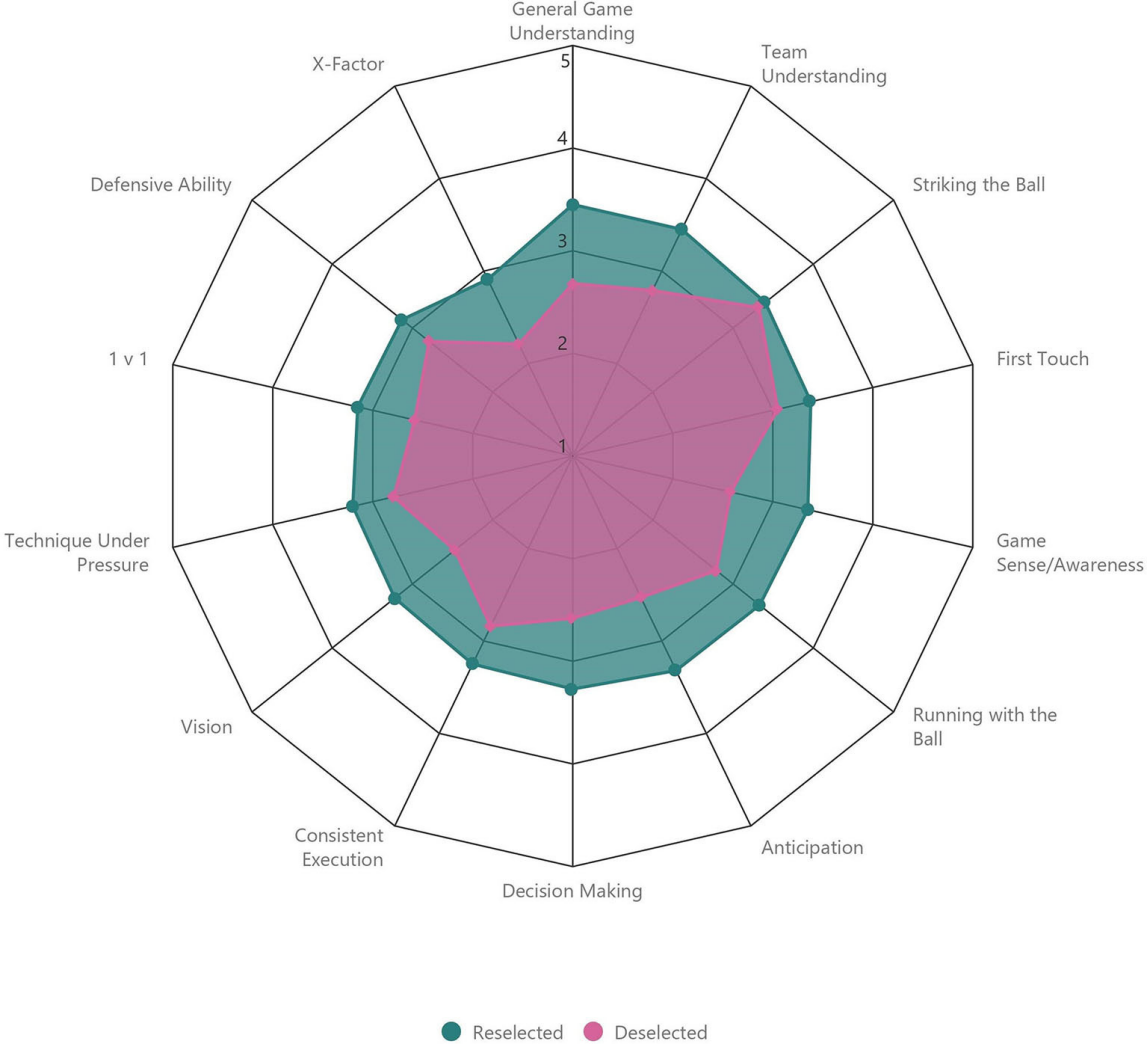
Comparison of coach ratings for attributes categorized as “Skill” between “Reselected” and “Deselected” players.

**Figure 3:**
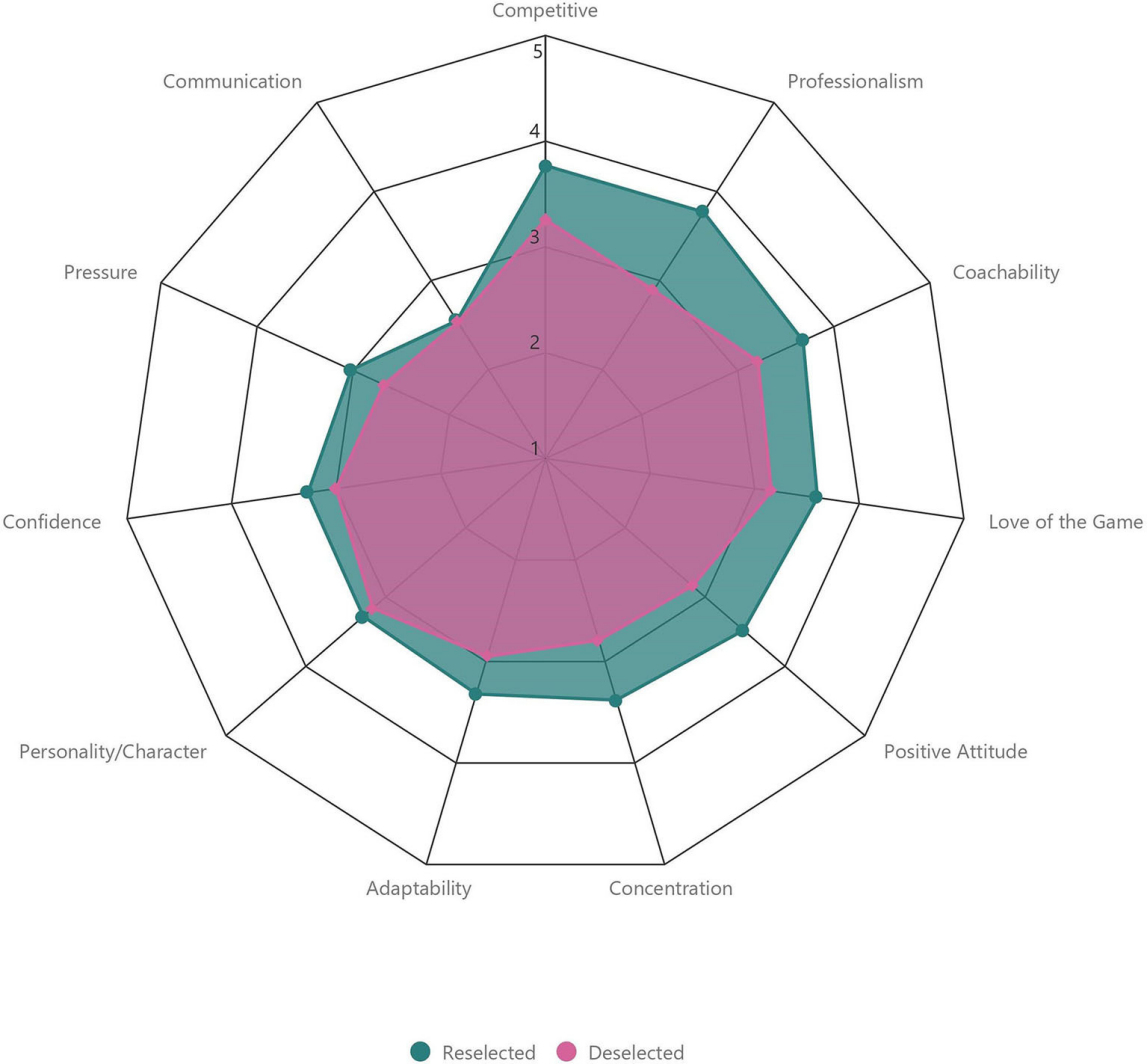
Comparison of coach ratings for attributes categorized as “Psychological” between “Reselected” and “Deselected” players.

**Figure 4:**
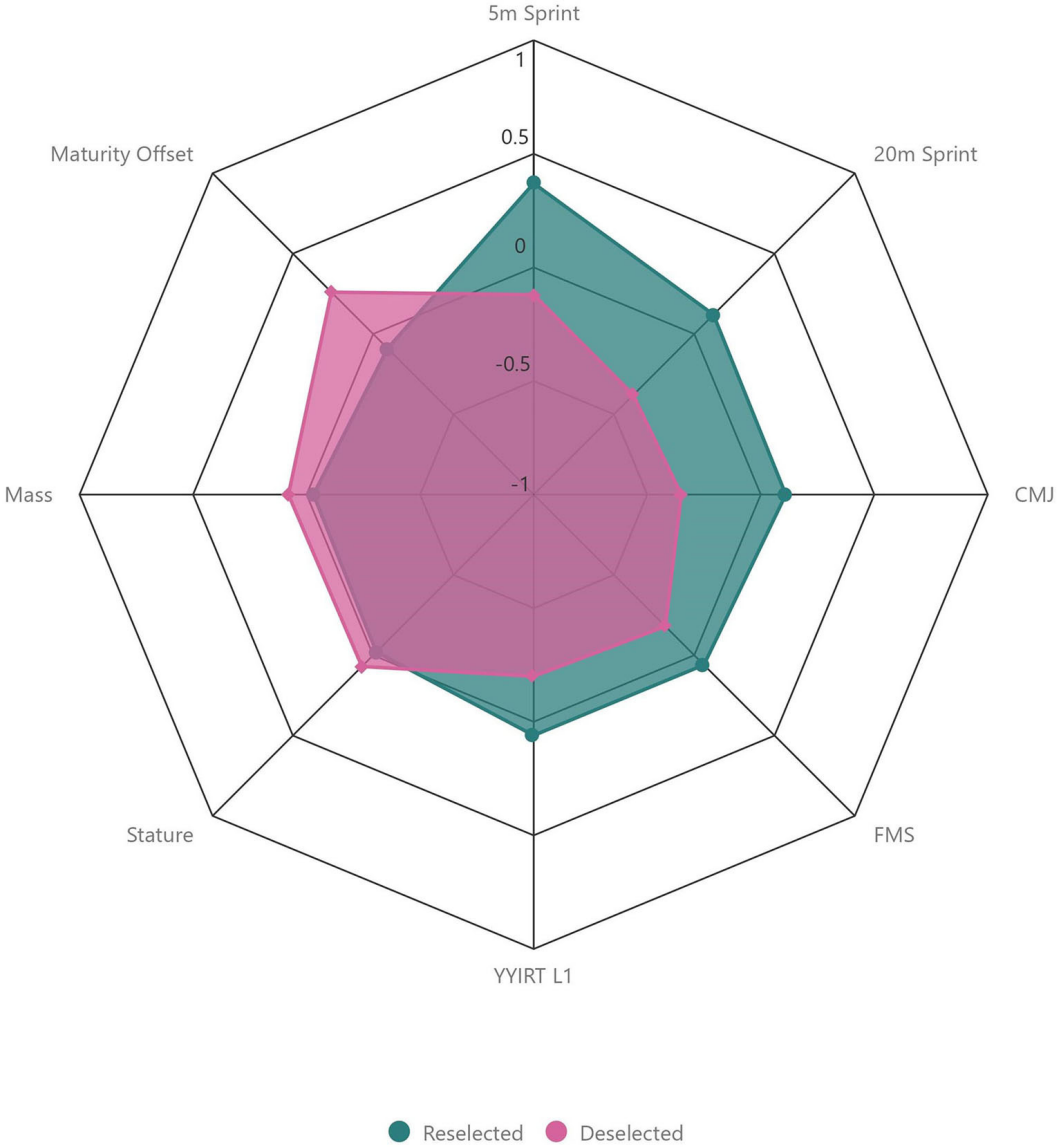
Comparison of objective physical data between “Reselected” and “Deselected” players. Data are presented as z-scores standardized by age group.

“Reselected” players were rated higher than “Deselected” players by coaches for all attributes within the “Skill” category (Table 1, Figure 2). The largest difference in rating between “Reselected” and “Deselected” players was observed between attributes of “General Game Understanding” (+0.8, *d* = 1.05; *moderate*), “Game Sense/Awareness” (+0.8, *d* = 1.06; *moderate*), and “Anticipation” (+0.8, *d* = 1.14; *moderate*). The smallest difference in rating was observed for “Striking the Ball” (+0.1, *d* = 0.14; *trivial*). “Reselected” players were also rated higher than “Deselected” players by coaches for all attributes within the “Psychological” category (Table 1, Figure 3). The largest difference in rating between “Reselected” and “Deselected” players was observed for “Professionalism” (+0.9, *d* = 1.05; *moderate*). The smallest difference in rating was observed between attributes of “Personality/Character” (+0.1, *d* =0.10; *trivial*) and “Communication” (+0.1, *d* = 0.09; *trivial*). When examining “Physical” attributes standardized by age group, “Reselected” players performed better than “Deselected” players on all fitness tests. The largest difference in fitness test performance between “Reselected” and “Deselected” players was observed between “CMJ” (+0.5, *d* = 0.51; *small*), “5 m Sprint” (+0.5, *d* = 0.54; *small*), and “20 m Sprint” (+0.5, *d* = 0.53; *small*). However, “Reselected” players were lesser in “Mass” (−0.1, *d* = 0.11; *trivial*), “Stature” (−0.1, *d* = 0.09; *trivial*), and “Maturity Offset” (−0.35, *d* = 0.39; *small*) than “Deselected” players (Table 1, Figure 4).

**Table 1:** Comparison between “Reselected” and “Deselected” players for coach subjective ratings of attributes categorized as “Skill,” “Psychological,” and “Physical.”

		**Reselected**	**Deselected**	**Effect size**	
		**(*n* = 60)**	**(*n* = 19)**	**(*****d*****)**	
Skill	First touch	3.4 ± 0.7	3.1 ± 0.8	0.40	Small
	Striking the ball	3.4 ± 0.7	3.3 ± 0.7	0.14	Trivial
	1 v 1	3.2 ± 0.8	2.6 ± 0.7	0.80	Moderate
	Decision making	3.3 ± 0.8	2.6 ± 0.7	0.93	Moderate
	Technique under pressure	3.2 ± 0.9	2.8 ± 0.4	0.57	Small
	Running with the ball	3.3 ± 0.9	2.8 ± 0.8	0.59	Small
	X-Factor	2.9 ± 1.1	2.2 ± 0.7	0.76	Moderate
	General game understanding	3.5 ± 0.9	2.7 ± 0.6	1.05	Moderate
	Game sense/awareness	3.4 ± 0.8	2.6 ± 0.7	1.06	Moderate
	Anticipation	3.3 ± 0.7	2.5 ± 0.7	1.14	Moderate
	Consistent execution	3.3 ± 0.8	2.8 ± 0.4	0.79	Moderate
	Vision	3.2 ± 0.6	2.5 ± 0.7	1.07	Moderate
	Team understanding	3.5 ± 0.8	2.8 ± 0.5	1.05	Moderate
	Defensive ability	3.1 ± 0.9	2.8 ± 0.8	0.35	Small
Psychological	Coachability	3.6 ± 1.0	3.2 ± 1.0	0.40	Small
	Positive attitude	3.5 ± 1.1	2.8 ± 0.9	0.70	Moderate
	Love of the game	3.6 ± 1.1	3.2 ± 0.8	0.42	Small
	Confidence	3.3 ± 0.9	3.0 ± 0.9	0.33	Small
	Competitive	3.8 ± 1.0	3.3 ± 0.8	0.55	Small
	Personality/character	3.3 ± 1.1	3.2 ± 0.8	0.10	Trivial
	Adaptability	3.3 ± 0.9	3.0 ± 0.5	0.41	Small
	Concentration	3.4 ± 1.0	2.8 ± 1.0	0.60	Moderate
	Professionalism	3.8 ± 1.1	2.9 ± 0.5	1.05	Moderate
	Communication	2.6 ± 1.2	2.5 ± 0.9	0.09	Trivial
	Pressure	3.0 ± 0.8	2.7 ± 0.6	0.42	Small
Physical	Mass	−0.03 ± 0.9	0.08 ± 1.1	0.11	Trivial
	Stature	−0.02 ± 1.0	0.07 ± 0.9	0.09	Trivial
	Maturity offset	−0.09 ± 1.0	0.26 ± 0.8	0.39	Small
	FMS	0.06 ± 0.9	−0.18 ± 1.1	0.24	Small
	CMJ	0.11 ± 1.0	−0.35 ± 0.8	0.51	Small
	5 m Sprint	0.37 ± 1.0	−0.12 ± 0.8	0.54	Small
	20 m Sprint	0.12 ± 1.0	−0.38 ± 0.9	0.53	Small
	YYIRT L1	0.06 ± 1.0	−0.2 ± 0.9	0.27	Small

“*Skill” and “Psychological” data are presented as Mean ± SD, “Physical” data are presented as within-group z-scores standardized by age group and presented as Mean ± SD*.

The frequency and percentage distributions of players' birth quartiles for both “Reselected” and “Deselected” player groups are presented in Table 2. Players born in Q1 and Q2 were overrepresented for both “Reselected” and “Deselected” player groups. The Chi-squared statistic also demonstrated significant deviations across quartiles for both “Reselected” and “Deselected” player groups.

**Table 2:** Birth quartile distributions for “Reselected” and “Deselected” players.

		**Birthdate distribution (%)**				**Odds ratio (95% CI)**			**Chi-squared**
**Playing level**	***n***	**Q1**	**Q2**	**Q3**	**Q4**	**Q1 vs. Q4**	**Q2 vs. Q4**	**Q3 vs. Q4**	**χ^2^**
Reselected	60	18 (30.0)	25 (41.7)	9 (15.0)	8 (13.3)	2.3 (1.0–5.3)	3.1 (1.4–7.2)	1.1 (0.5–2.8)	21.6^*^
Deselected	19	7 (36.8)	7 (36.8)	3 (15.8)	2 (10.5)	3.5 (1.5–8.5)	3.5 (1.5–8.5)	1.5 (0.6–3.9)	22.9^*^

* *Significant at an alpha level of p < 0.05. Q1 = Jan-Mar; Q2 = Apr-Jun; Q3 = Jul-Sep; Q4 = Oct-Nov*.

## Discussion

This exploratory case study examined differences in multidimensional attributes of players related to persistence/progression (“Reselected”) or attrition (“Deselected”) in playing level following closure of a youth soccer academy. The majority of the players within our study (76%) were “Reselected” to a junior-elite academy in the subsequent 6 months after being released. Our findings also showed that “Reselected” players were rated higher during subjective coach evaluations across all attributes within this study which were categorized as “Skill” or “Psychological.” We observed that “Reselected” players performed better than “Deselected” players in all objective physical fitness tests within our study; however, “Reselected” players were lesser in “Mass,” “Stature,” and “Maturity Offset” (further away from peak height velocity) than “Deselected” players. Finally, a similar asymmetry in birthdate distribution was observed for both “Reselected” and “Deselected” players favoring those born in the first half of the selection year.

The finding that the majority of our sample were re-signed in the 6 months following the academy closure supports the notion that individuals released from TD programmes are often recruited by rival clubs (Vaeyens et al., 2008; Unnithan et al., 2012). Coaches and practitioners attempt to identify and detect relevant characteristics of future soccer performance as early as possible through TI processes (Figueiredo et al., 2014). Moreover, prolonged exposure to systematic TD is considered crucial to successful reselection and youth-professional transition (Williams and Reilly, 2000; Baker et al., 2012). The players within our study were released from their contracts due to closure of the soccer academy, opposed to being deselected for performance reasons. Therefore, these players may have been more appealing to rival academies than amateur players due to their prior selection to, and engagement in, a systematic TD programme. Scouting processes traditionally inform selection and recruitment decisions in academy soccer (Reeves and Roberts, 2019; Williams et al., 2020). Similarly, coaches and recruiters have been shown to engage in informal evaluation processes of players during competition (Reeves et al., 2019). Therefore, it is possible that rival academies may have been familiar with the players within our study and may have had preconceived subjective opinions of their abilities prior to their release. It is also possible that as a duty of care, the Academy Director and age group coaches may have utilized their network to assist players in continuing to play academy-level soccer. Such processes have previously been reported by deselected athletes across other sports (Williams and MacNamara, 2020). We propose that previous exposure to a TD programme may explain the prevalence of reselection we observed. However, we acknowledge the potential sociological factors that may have influenced (de)selection and recruitment decisions within our study.

Our findings evidence no age-related trends in persistence/progression or attrition in playing level following the academy closure. The accumulation of appropriate practice hours is deemed crucial to successful transition to professional soccer (Ford and Williams, 2012; Haugaasen et al., 2014). However, high turnover of youth players is reported within professional soccer academies, with only ~10% of players successful in obtaining professional contracts (Grossmann and Lames, 2015). The probability of successful youth-professional transition is increased in the latter years of youth soccer academy development (Kannekens et al., 2011). Yet, we observed comparable reselection rates at U13 and U17, and U12 and U15 age groups, respectively. When observing age category transitions for German junior-elite academy soccer players, 67–83% of players were successful in being selected to the subsequent age group (Güllich, 2014). Consequently, reselection rates observed in our study (reselection to another academy TD programme due to non-performance-related deselection) may be more comparable to age category transitions than traditional deselection/reselection observations.

“Reselected” players received a higher overall subjective coach category rating than “Deselected” players for “Skill,” “Psychological,” and “Physical” abilities during our study. Both “Skill” and “Psychological” attributes are identified as important by coaches and recruiters when making decisions around (de)selection in youth soccer (Larkin and O'Connor, 2017; Roberts et al., 2019). However, these authors suggest that although coaches and recruiters deem physical abilities necessary for soccer, they may value them less than skill or psychological attributes. A number of longitudinal studies have reported that future professional players perform better and receive higher coach ratings for both skill (Van Yperen, 2009; Forsman et al., 2016; Höner et al., 2017; Sieghartsleitner et al., 2019) and psychological (Gledhill et al., 2017; Murr et al., 2018a) attributes. Yet, despite lower perceived importance by coaches and recruiters, future professional players also exhibit greater physical performance when compared to non-professional players (Gravina et al., 2008; le Gall et al., 2010; Gonaus and Müller, 2012; Emmonds et al., 2016). Differing approaches to talent identification and selection/deselection processes have been observed in soccer, largely influenced by varied philosophies held by coaches or clubs, or by the perceived competition demands of the nation or league observed (Unnithan et al., 2012; Reeves et al., 2018a). We observed similar differences between “Reselected” and “Deselected” players for all overall category ratings. Therefore, our results suggest that, in a Scottish context, “Skill,” “Psychological,” and “Physical” abilities may be of similar importance to TI and the (de)selection process in soccer.

When examining coach ratings for attributes categorized within “Skill,” we observed the largest differences between “Reselected” and “Deselected” players for “Game Understanding,” “Game Sense/Awareness,” and “Anticipation,” and we observed the smallest difference for the rating of “Striking the Ball.” Attributes we observed as the largest differences between groups have previously been categorized as “perceptual-cognitive skills” in soccer (Roca et al., 2012; Williams et al., 2012; Roberts et al., 2019). Perceptual-cognitive skills typically refer to the ability of performers to identify and process environmental information for integration with existing knowledge to facilitate the selection of appropriate responses under time pressure (Williams, 2000; Williams et al., 2012). Acknowledging the intermittent and high intensity demands of soccer competition (Di Salvo et al., 2009), perceptual-cognitive abilities have been championed when comparing skilled and less skilled soccer players (Williams, 2000; Williams et al., 2020). On the contrary, scientists report that technical skills performed in isolation are least representative of *in-situ* performance (Williams and Reilly, 2000; Unnithan et al., 2012), perhaps explaining the small difference we observed between “Reselected” and “Deselected” players for the rating of “Striking the Ball.” Our results reiterate the importance of multidimensional opposed to isolated skill qualities in soccer, with a particular emphasis on the (de)selection process.

Coach ratings of “Professionalism” demonstrated the largest difference between “Reselected” and “Deselected” players for attributes categorized as “Psychological.” Within soccer, “Professionalism” may encompass a range of player behaviors during both training and competition, such as conduct, mannerisms, and autonomy (Martindale et al., 2007; Larkin and O'Connor, 2017). Considering that “Professionalism” is not explicitly identified when identifying potential psychological predictors in soccer (Höner and Feichtinger, 2016; Murr et al., 2018a), the definition of “Professionalism” we provided to coaches (Larkin and O'Connor, 2017) may have been vaguely interpreted, potentially capturing elements of wider psychological attributes. On the contrary, the smallest difference in coach ratings between “Reselected” and “Deselected” players was observed between attributes of “Personality/Character” and “Communication.” Interestingly, elements of the definitions provided to coaches for these two attributes are similar to the definition of “Professionalism” (Larkin and O'Connor, 2017). In light of these observations, we suggest that a degree of ambiguity and individual interpretation may have occurred during the coach rating process. Despite presenting previously identified attributes and definitions related to the soccer recruitment process in soccer, we suggest that, in line with recent work, predictors may need to be identified and established by the coaches in a two-part process (Reeves et al., 2018b; Roberts et al., 2019).

Finally, the greatest difference in objective “Physical” fitness performance between “Reselected” and “Deselected” players was observed for the “CMJ,” “5 m Sprint,” and “20 m Sprint” tests. Neuromuscular qualities, such as speed and power, receive particular interest during TI and TD compared to other physical attributes (Murr et al., 2018b). Furthermore, soccer players playing at a higher competitive level often outperform those playing at a lower competitive level on CMJ and sprint tests (Coelho E Silva et al., 2010; le Gall et al., 2010; Dugdale et al., 2019). We suggest that as a result of the recent increases in physical demands of adult soccer match play (Barnes et al., 2014; Bush et al., 2015), differences in speed and power performance may provide useful information to contribute to TI and (de)selection decision making in soccer.

Despite outperforming “Deselected” players during physical fitness tests, “Reselected” players were lesser in “stature,” “mass,” and “maturity offset” than “Deselected” players within our study. Typically, relationships are observed between more mature players of larger body size and physical performance (see Kelly and Williams, 2020 for a review). However, exceptions to this observation have been reported (Reilly et al., 2000; Malina et al., 2007; Figueiredo et al., 2009; Deprez et al., 2014). Furthermore, anthropometric profiles have been suggested to be position-specific, with physical attributes being favorable for certain positions (Gil et al., 2007; Deprez et al., 2014). A wealth of evidence suggests that adolescent soccer players may be selected to TD programmes due to superior anthropometric profiles and maturity status (Malina et al., 2017; Kelly and Williams, 2020). Yet, this selection bias lacks efficacy (Burgess and Naughton, 2010; Meylan et al., 2010). Our observations that “Deselected” players were taller, heavier, and more mature than “Reselected” players further support this premise. We also observed a prevalent asymmetry in birthdate distribution for our entire sample, but no difference between “Reselected” and “Deselected” players. This suggests that recruitment to the academy in the first instance may have conformed to these aforementioned biases (Deprez et al., 2014; Castillo et al., 2019); however, birth month did not influence (de)selection of players within our study.

Our study is not without limitations. One limitation of the present study relates to the sample size and disparity between “Reselected” and “Deselected” player groups. In light of this limitation, we present a novel yet highly relevant case study following the closure of a junior-elite soccer academy. Although a greater sample size and ability to explore differences between groups would strengthen this study, the inability to control these factors and novelty of this situation must be acknowledged. As a result, we encourage readers of our study to treat our results with appropriate caution given the design utilized. Further research should attempt to identify larger samples for a more thorough comparison between “Reselected” and “Deselected” players including sub-analyses for factors such as age and playing position. Secondly, we must acknowledge the subjective nature of ratings for some of the attributes within our study, as opposed to objective measures of performance previously used to evaluate differences between reselected and deselected players (Figueiredo et al., 2009; Huijgen et al., 2014). Deriving attribute ratings via subjective methods is typical of traditional scouting methods utilized during the recruitment and selection/deselection processes of youth soccer players, therefore relevant to applied practice (Reeves and Roberts, 2019). We suggest that readers acknowledge the potential limitations associated with this rating method when interpreting our results (Dugdale et al., 2020).

## Practical Implications

Our findings reiterate the longstanding notion that potential predictors associated with TI and TD in soccer should be multidimensional in nature, and attempt to progress the repeated call for more multidisciplinary research in soccer. Furthermore, we encourage the use of both subjective and objective data to provide a time and resource efficient method of gathering information that may be of value to decision making during (de)selection processes. The visualization methods implemented in this study (radar plots) may assist when presenting a large volume of multidisciplinary data. This may assist coaches and recruiters to better understand strengths and weaknesses of youth soccer players but also visually convey compensatory mechanisms (i.e., superior strengths that counteract weaknesses in other areas) that may previously have been overlooked. Finally, given the constant and increasing financial pressures placed on professional football clubs, we provide a case study approach of a unique yet highly relevant scenario for talent identification and development in youth soccer.

## Conclusion

In summary, the majority of players persisted or progressed in playing level following closure of a junior-elite soccer academy. These players were rated higher by coaches for all “Skill” and “Psychological” attributes, as well as performing better in “Physical” fitness tests. However, anthropometric and maturity variables were more pronounced for players who experienced an attrition in playing level. Our findings further support the multidisciplinary nature of talent in soccer and promote the requirement for multiple assessment methods when making (de)selection decisions during TI. Moreover, we encourage coaches and recruiters to acknowledge the potential limitations of making (de)selection decisions influenced only by physical factors related to growth and maturation.

## Data Availability Statement

The original contributions presented in the study are included in the article/supplementary material, further inquiries can be directed to the corresponding author/s.

## Ethics Statement

The studies involving human participants were reviewed and approved by University of Stirling cross-faculty ethics committee. Written informed consent to participate in this study was provided by the participants' legal guardian/next of kin.

## Author Contributions

JD, AM, and VU contributed to the conception and design of the study. JD performed the data collection and wrote the first draft of the paper. JD and AM performed the data analysis. All authors contributed to manuscript revision and read and approved the submitted version.

## Conflict of Interest

The authors declare that the research was conducted in the absence of any commercial or financial relationships that could be construed as a potential conflict of interest.
